# Lifestyle Screening Tools for Children in the Community Setting: A Systematic Review

**DOI:** 10.3390/nu14142899

**Published:** 2022-07-14

**Authors:** Anne Krijger, Sovianne ter Borg, Liset Elstgeest, Caroline van Rossum, Janneke Verkaik-Kloosterman, Elly Steenbergen, Hein Raat, Koen Joosten

**Affiliations:** 1 Department of Pediatrics and Pediatric Surgery, Erasmus MC-Sophia Children’s Hospital, University Medical Center Rotterdam, 3000 CB Rotterdam, The Netherlands; k.joosten@erasmusmc.nl; 2 Department of Public Health, Erasmus MC, University Medical Center Rotterdam, 3000 CA Rotterdam, The Netherlands; l.elstgeest@erasmusmc.nl (L.E.); h.raat@erasmusmc.nl (H.R.); 3 National Institute for Public Health and the Environment, 3720 BA Bilthoven, The Netherlands; sovianne.ter.borg@rivm.nl (S.t.B.); caroline.van.rossum@rivm.nl (C.v.R.); janneke.verkaik@rivm.nl (J.V.-K.); elly.steenbergen@rivm.nl (E.S.); 4 Reinier Academy, Reinier de Graaf Hospital, 2600 GA Delft, The Netherlands

**Keywords:** screeners, nutrition, physical activity, sedentary behaviour, lifestyle risk, obesity

## Abstract

Screening of children’s lifestyle, including nutrition, may contribute to the prevention of lifestyle-related conditions in childhood and later in life. Screening tools can evaluate a wide variety of lifestyle factors, resulting in different (risk) scores and prospects of action. This systematic review aimed to summarise the design, psychometric properties and implementation of lifestyle screening tools for children in community settings. We searched the electronic databases of Embase, Medline (PubMed) and CINAHL to identify articles published between 2004 and July 2020 addressing lifestyle screening tools for children aged 0–18 years in the community setting. Independent screening and selection by two reviewers was followed by data extraction and the qualitative analysis of findings. We identified 41 unique lifestyle screening tools, with the majority addressing dietary and/or lifestyle behaviours and habits related to overweight and obesity. The domains mostly covered were nutrition, physical activity and sedentary behaviour/screen time. Tool validation was limited, and deliberate implementation features, such as the availability of clear prospects of actions following tool outcomes, were lacking. Despite the multitude of existing lifestyle screening tools for children in the community setting, there is a need for a validated easy-to-administer tool that enables risk classification and offers specific prospects of action to prevent children from adverse health outcomes.

## 1. Introduction

A healthy lifestyle is essential for optimal growth and development as well as for later-life health of children [1,2]. The World Health Organization proposed the concept of a healthy lifestyle to be ‘a way of living that lowers the risk of being seriously ill or dying early’ [3]. A large number of factors can be considered as lifestyle. In children, nutrition, physical activity (PA), sedentary behaviour and sleep are lifestyle factors that were found to be associated with health outcomes [4,5,6,7]. Overweight, obesity and other cardiovascular risk factors are common consequences of an unhealthy lifestyle and may already appear during childhood [4]. The adequate evaluation of children’s lifestyle can contribute to preventive actions that combat the increasing prevalence of lifestyle-related conditions. 

To evaluate the lifestyle of children, including nutrition, various tools can be used. Two groups of lifestyle tools can be distinguished: lifestyle assessment tools and lifestyle screening tools [8]. Lifestyle assessment tools, such as food frequency questionnaires, 3-day food diaries and physical activity trackers, are used to examine the child’s behaviour and/or characteristics in detail. To be of service to youth healthcare, which has a preventive function but limited consultation time, this paper focuses on lifestyle screening tools that identify risk (factors) on an individual level. Lifestyle screening tools usually comprise more general items than lifestyle assessment tools, are used for quick evaluation and assign a certain value to the lifestyle behaviour and/or characteristics of the child. In practice, a commonly used method for this is a short questionnaire. Outcomes of lifestyle screening tools may vary; they can, for example, result in an overall lifestyle score or highlight areas for improvement (‘red flags’). Given the rapid value judgment, lifestyle screening tools can be helpful in clinical practice or community screening. Here, they can serve as a basis to enter into dialogue with the parents or provide advice for further actions, for instance, referral to a dietitian or starting an intervention. Next to the design characteristics of lifestyle screening tools (such as the number of items, covered topics and intended target group), the psychometric properties (i.e., reliability and validity) and implementation methods (such as the manner in which the outcomes or advice for further action are formulated (prospects of action)), practical application and tool format (online, on paper, etc.) are likely to affect the usability and effectiveness of such screening tools.

Reviews specifically on nutrition screening tools for children have mainly focused on tools developed for hospital settings [9,10,11,12,13]. A recently published systematic review by Becker et al. targeted the reliability and validity of nutrition screening tools for children up to 18 years of age, including tools for the community setting [14]. The community health care setting, represented by preventive and primary health care services, is the perfect place for the usage of lifestyle screening tools. This is because most children with a suboptimal lifestyle reside in the community setting and will not be admitted to a hospital. A thorough overview of existing lifestyle screening tools for children aged 0–18 years in the community setting, not limited to nutrition, is yet lacking. 

Therefore, our systematic review aims to comprehensively describe lifestyle screening tools for children in the community setting. The present study is embedded in a Dutch governmental project that intends to develop a lifestyle screening tool for children aged 0–4 years. This screening tool will ultimately lead to timely measures to prevent children from negative lifestyle-related health outcomes. The specific questions to be addressed within our review are:

(1) What lifestyle screening tools for children in the community setting are available?
(2) What are the main features of these lifestyle screening tools regarding design, psychometric properties (i.e., reliability and validity) and implementation?

## 2. Materials and Methods

This systematic review is reported as indicated in the Preferred Reporting Items for Systematic reviews and Meta-Analyses (PRISMA) guideline [15]. An a priori systematic review protocol was developed (available upon request). 

### 2.1. Search Strategy

We performed systematic searches in the electronic databases of Embase, Medline (PubMed) and CINAHL to identify articles addressing lifestyle screening tools for children in the community setting, published between January 2004 and July 2020. Based on the study objectives, the PICO model [16] was used to further specify the search strategy. The population (P) was defined as children up to 18 years of age in the community setting, the intervention/exposure (I) as lifestyle screening tools and the outcomes of interest (O) as indicators of an unhealthy lifestyle. We did not include a comparison to a control group (C) as we did not study an intervention effect. Search strings were developed with assistance from a librarian. Search terms were divided into the categories ‘child’, ‘screening’ and ‘lifestyle’, which were combined with ‘AND’. Emtree terms and MeSH terms were used to identify relevant articles (Supplementary file S1). Search filters to restrain the results to humans and English or Dutch language were applied. The search strategies were not limited to specific lifestyle factors.

As nutrition is such an eminent part of lifestyle, we performed additional literature searches focusing on nutrition screening tools. Hence, we updated the searches by Becker et al. and an exploratory systematic search that was conducted in 2019 (unpublished research, for details, see Supplementary file S1). Similar to the broader search on lifestyle screening tools, filters to limit the results to humans and English or Dutch language were applied.

Full details on the search strings are provided in Supplementary file S1. Search results were exported to EndNote X9 reference management software and deduplicated.

### 2.2. Eligibility Criteria

For the inclusion of an article, the following predefined criteria had to be met:

1. The study described a screening tool to identify lifestyle risk (factors) on an individual level for
2. children up to 18 years of age in
3. the community setting.
4. The tool had to be applied by a parent/caregiver, health professional (e.g., physician, nurse) or by the child him- or herself, and
5. the study was published in English or Dutch
6. between January 2004 and July 2020.

Exclusion criteria comprised:

1. studies reporting on lifestyle questionnaires, with a purpose other than screening for lifestyle risk (factors) on an individual level (e.g., general questionnaires in national surveys);
2. studies on lifestyle assessment tools (e.g., (derivatives of) food frequency questionnaires, diet quality scores, anthropometry);
3. studies on a single specific lifestyle or nutrition factor (e.g., solely screen time or vegetable intake);
4. studies reporting prevalence rates of malnutrition or growth charts as a measure of nutrition risk;
5. tools to identify eating disorders;
6. tools developed for hospital settings or specific patient groups;
7. commentaries and conference abstracts.

### 2.3. Screening, Selection and Data Extraction

Applying the abovementioned inclusion and exclusion criteria, two reviewers (A.K. and S.t.B.) independently screened titles and abstracts of the obtained articles. Thereafter, they selected the relevant articles based on full texts according to the same inclusion and exclusion criteria. Additionally, articles included in the review of Becker et al. [14] and identified with the exploratory search on nutrition screening tools were checked for eligibility. Discrepancies in opinion on inclusion by the reviewers were resolved by discussion until consensus or in consultation with a third reviewer (L.E.). A.K. and S.t.B. then extracted the data from the included studies. Reported general information (reference, title), study characteristics (study objective, study year, country of origin, study design, sample size, age, outcome measures, results) and tool characteristics (tool name, tool aim, target population, person administered, administer duration, administer frequency, administer method, addressed domains, number of items, response format, tool outcome, prospect of action, strengths, limitations) were entered into a predesigned data extraction table. The usability of the data extraction table was tested beforehand by extracting data from 10% of the articles in duplicate by A.K. and S.t.B.. Articles reporting on the same tool were grouped. Articles covered in included reviews were also assessed for eligibility.

### 2.4. Data Analysis

By summarising the characteristics of the included studies and corresponding lifestyle screening tools, we performed an initial data synthesis. Subsequently, qualitative analysis was performed by tabulating and assorting by specific features, such as target age (toddlers, 1–3 years old; preschoolers, 3–5 years old; school age, 6–12 years old; adolescents, 13–18 years old), number of tool items and prospects of action. This enabled us to aggregate the data further and to explore similarities and differences between the identified screening tools.

## 3. Results

**Figure 1:**
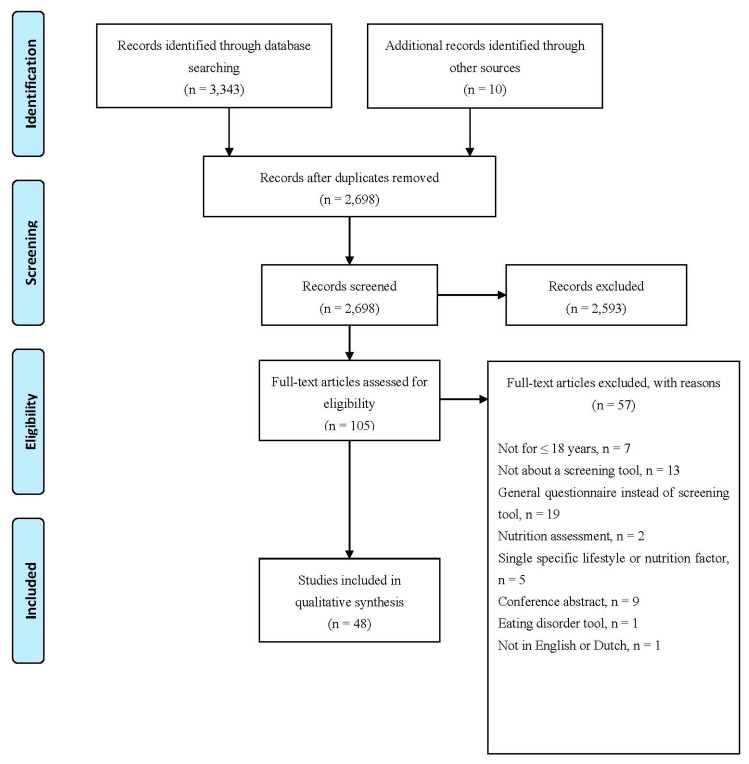
PRISMA flowchart of methodology.

A total of 2698 articles were identified for screening (Figure 1). After the full-text review of 105 articles, 48 met the inclusion criteria and were included in the qualitative analysis. The most common reasons for exclusion were: not describing a screening tool or describing a general questionnaire instead of a screening tool. We included two systematic reviews [14,17], yielding no additional screening tools for inclusion. The other 46 articles [18,19,20,21,22,23,24,25,26,27,28,29,30,31,32,33,34,35,36,37,38,39,40,41,42,43,44,45,46,47,48,49,50,51,52,53,54,55,56,57,58,59,60,61,62,63] described 41 unique screening tools. The majority of the included articles reported on the development and validation of screening tools, whereas their implementation was rarely addressed. Studies were performed between 2001 and 2019 in sixteen different countries (both Western and non-Western), with nearly half conducted in the United States (*n* = 20). 

### 3.1. Design of Screening Tools

Table 1 demonstrates various characteristics of the included lifestyle screening tools. The majority of tools were developed to screen lifestyle behaviour and habits. Although not always explicitly stated in the tool’s aim, articles mostly described that the tool focused on factors associated with obesity risk. Ten screening tools were distinctively designed for toddlers (1–3 years old) or preschoolers (3–5 years old) [18,19,20,21,22,23,24,25,26,27,28,29,30,31] and another nine for school-aged children (6–12 y) [32,33,34,35,36,37,38,39]. Fourteen tools were described as either designed for children in general or did not specify the children’s target age (0–18 y) [40,41,42,43,44,45,46,47,48,49,50,51,52,53,54,55]. Eight tools were specifically designed for adolescents (13–18 y) [56,57,58,59,60,61,62,63]. The tools aimed at toddlers and preschoolers were to be administered by parents or health care professionals. Children of school age reported themselves (*n* = 6) or their parents did (*n* = 3). One tool for children without specified age was divided into a part completed by the child and a part completed by the parents [55]. Tools for adolescents only were exclusively self-reported. Tools administered to parents could include proxy-reported items on the child but also self-reported items regarding parents themselves, such as self-efficacy for a healthy lifestyle or parental feeding practices. The number of items per tool ranged from 3 to 116, with a median of 22 items (interquartile range (IQR): 17, 34). No article described the rationale for the number of items. All tools used multiple choice questions (some combined with open questions), mainly on Likert-type scales. Two tools used visuals to increase comprehensibility [30,37]. These visuals included portion sizes and images to make the tool more appealing. The time needed to complete the tool was reported for only thirteen tools [18,19,20,30,31,34,37,38,39,40,47,52,60,63]. From those who reported the time, the time needed ranged from 3 [18,19,20] to 90 [37] minutes; six tools could be completed within 15 min [18,19,20,31,38,40,52]. 

**Figure 2:**
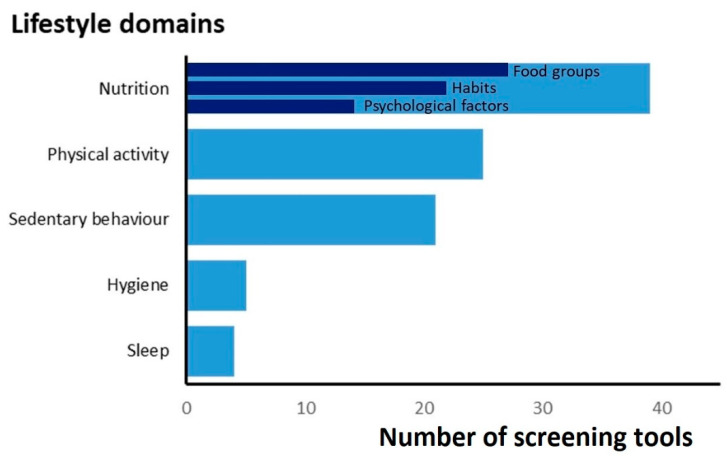
Prevalence of most frequently covered domains. N.B. The total number of covered domains exceeds the number of screening tools (*n* = 41) since most tools covered multiple domains.

Table 2 shows the encompassed lifestyle domains with specified items of the included screening tools. Specification of the nutrition items is demonstrated in Table 3. The domains covered most were nutrition (*n* = 39), PA (*n* = 25) and sedentary behaviour/screen time (*n* = 21) (Figure 2). The median of the number of covered domains was three. Tools for toddlers and preschoolers covered, with a median of two, fewer domains. All screening tools intended for toddlers and preschoolers covered nutrition. None of the screening tools specifically for toddlers included PA items, whereas, in other tools, PA was mainly evaluated by estimating the frequency and duration per week. Sedentary behaviour was not determined as such but evaluated with screen time as proxy. Sleep and hygiene were included in four and five tools, respectively, mainly as sleep duration (*n* = 2) and dental care (*n* = 4). Huang et al. included neighbourhood safety [55]; environmental factors in other tools were generally related to nutrition and PA (e.g., parental modelling). As for the items on nutrition, the intake of specific food groups, dietary habits and psychological factors were predominantly evaluated (Table 3). Of all the tools that evaluated the consumption of food groups (*n* = 27), most asked about vegetables (*n* = 25), fruits (*n* = 25), sugar-sweetened beverages (*n* = 16) and unhealthy snacks/fast food (*n* = 16). Commonly addressed eating habits were consuming breakfast (*n* = 9), eating at the table or while watching TV (*n* = 6) and eating with the family together (*n* = 5). Psychological factors mainly included (parental) beliefs and attitudes towards healthy eating. In addition, nutrition knowledge (*n* = 4) and food costs (*n* = 2) recurred in several tools.

**Table 1:** Characteristics of lifestyle screening tools for children in the community setting.

Tool Name	Tool Aim	Target Population	Administered by	Number of Items	Item Response Format	Tool Scoring	Prospect of Action
1. NutricheQ [18,19,20]	Assess dietary risk	Toddlers	Parent ^b^	11 ^c^	3-point Likert scale	Subsection score and total score; ranging from 0 to 22 Cut-offs for low, moderate and high risk are available per section	Tool identifies children who may need blood screening and nutritional intervention
2. Toddler Feeding Questionnaire (TFQ) [21]	Assess indulgent, authoritative and environmental feeding practices	Toddlers	Parent ^a^	24	5-point Likert scale (never-always)	Subscale scores	NR
3. Toddler NutriSTEP [22]	Assess nutritional risk	Toddlers	Parent ^b^	17	Likert-type scale	Total score; ranging from 0 to 68 Cut-offs for low, moderate and high risk	Treat impaired state and refer to needed services
4. Toddler Dietary Questionnaire (TDQ) [23]	Assess dietary risk	Toddlers	Parent ^b^	19	Likert-type scale	Total score; ranging from 0 to 100 Cut-offs for low, moderate, high and very high dietary risk	Health care professionals may refer to a dietitian based on identified risk
5. Child Eating Behavior Questionnaire (CEBQ) [24]	Assess eating behaviours	Preschoolers	Parent ^b^	35	5-point Likert scale (never-always)	Subscale scores	NR
6. Nutrition Screening Tool for Every Preschooler (NutriSTEP) [25,26,27]	Assess nutritional risk	Preschoolers	Parent ^b^	17	Likert-type scale (varying)	Total score; ranging from 0 to 68 Cut-offs for low, moderate and high risk	Parents receive results, customised feedback and resources such as links to credible health information websites
7. Preschooler Dietary Questionnaire (PDQ) [28]	Assess diet and provide a dietary risk score	Preschoolers	Parent ^b^	19	Likert-type scale	Total score; ranging from 0 to 100 Cut-offs for low, moderate, high and very high dietary risk	Health care professionals may refer to a dietitian based on identified risk
8. Preschoolers Diet–Lifestyle Index (PDL-index) [29]	Assess adherence to diet–lifestyle recommendations	Preschoolers	Health care professional ^b^	11	Likert-type scale (varying)	Total score; ranging from 0 to 44	Tool may guide health care professionals in counselling parents and policy makers in developing interventions
9. Healthy Kids [30]	Assess diet, lifestyle and parenting domains to determine obesity risk	Children aged 2–5 y from low-income families	Parent ^a,b^	19	Combination of closed and open questions	Total score; ranging from 19 to 95	Tool can be used to target counselling or nutrition education for families and to supplement physical examination
10. Tool by Das and Ghosh [31]	Assess nutrition knowledge	Children aged 3–6 y	Parent ^a^	32	Closed questions	Total score; ranging 0–32	NR
11. Start the Conversation 4–12 (STC-4–12) [32]	Assess and counsel nutrition and PA barriers and behaviours	Children aged 4–12 y	Parent ^a,b^	22	Likert-type scale (varying)	No score	The tool provides tips that serve as cues for action for parents and guide counselling by health care professionals
12. Healthy Families Survey [33]	Assess nutrition and PA behaviours	Elementary school children	Parent ^a,b^	45	Combination of closed and open questions	Subscale scores	NR
13. Knowledge, Attitudes and Habits (KAH-) questionnaire [34]	Assess knowledge, attitudes and habits towards a healthy lifestyle	Elementary school children	Child ^a^	48	3-point Likert scale	Subscale scores and total score; ranging from 0 to 96	NR
14. Parental Self-efficacy Questionnaire [35]	Assess parental self-efficacy for enacting healthy diet and PA behaviours in their children	Children aged 6–11 y	Parent ^a^	34	11-point Likert scale	Subscale scores and total score; ranging from 0 to 340	NR
15. Tool by Chacko and Ganesan [36]	Assess dietary gaps	School children aged 6–17 y	Child ^a^	10	2-point Likert scales (yes–no)	Total score; ranging from 0 to 10	Parents and children can receive corrective counselling on the identified gaps
16. Food, Health and Choices questionnaire (FHC-Q) [37]	Assess energy balance behaviours and related theory-based psychosocial determinants	Upper elementary school children	Child ^a^	116	Likert-type scale	Subscale scores	NR
17. Healthy Eating and Physical Activity Self-Efficacy Questionnaire for Children (HEPASEQ-C) [38]	Assess self-efficacy of healthy eating and PA	Upper elementary school children	Child ^a^	9	3-point Likert scale (there is no way I can do this–I believe I can do this)	Total score; ranging from 9 to 27	NR
18. Healthy Eating and Physical Activity Behavior Recall Questionnaire for Children (HEPABRQ-C) [38]	Assess recall of healthy eating and PA	Upper elementary school children	Child ^a^	10	Combination of closed and open questions	Total score; ranging from 0 to 21	NR
19. Eating Behavior Questionnaire for School Children [39]	Assess eating behaviours	School children	Child ^a^	23	5-point Likert scale (never-always)	Subscore per domain	NR
20. Tool by Drouin and Winickoff [40]	Assess health-related behavioural risk factors	Children aged 0–18 y	Parent ^b^	3	Closed questions	No score	Parents receive a handout with information about identified risk factors Health care professionals receive the survey results and an evidence-based suggested course of action
21. Child Nutrition and Physical Activity (CNPA) Screening Tool [41]	Assess behaviours that increase the risk of obesity	Children aged 2–18 y	Parent ^a,b^	22	4-point Likert scale and open questions	Subscores for generated readiness to change and perception factors only	Tool provides health care professionals means to start the conversation about a healthy lifestyle with parents
22. Electronic Kids Dietary Index (E-KINDEX) [42]	Assess food habits, dietary beliefs and practices related to obesity	Children	Child ^a^, parent ^b^ or health care professional ^b^	30	Likert-type scale (varying)	Subscale scores and total score; ranging from 1 to 87	In clinical practice, the score can be used as visual educational tool, provide continuous feedback and individual items may be used as specific goals for obesity status improvement
23. Family Health Behavior Scale (FHBS) [43]	Assess family eating and PA habits related to obesity	Children	Parent ^a,b^	27	5-point Likert scale (never-nearly always)	Subscale scores and total score	NR
24. Family Nutrition and Physical Activity (FNPA) screening tool [44,45]	Assess risk factors for overweight/obesity in the home environment	Children	Parent ^a,b^	20	4-point Likert scale (never-always)	Subscore per domain and total score	Korean version: based on scores, interventions such as counselling and education should be developed and provided
25. HABITS questionnaire [46]	Assess weight-related behaviours and intervention targets	Children	Child ^a^	19	Likert-type scale (varying)	Subscale scores	Tool can establish a dialogue about weight-related lifestyle behaviours between health care professional and families
26. Healthy Living for Kids Survey (HLKS) [47]	Assess healthy lifestyle perceptions and behaviours	Children	Child ^a^	59	Likert-type scale (varying)	Subscale scores and total score	Education of parents and children to redress inaccurate perceptions of a healthy lifestyle
27. HeartSmartKids (HSK) [48] (HeartSmartKids, LLC, Boulder, US)	Assess lifestyle habits to guide behaviour change counselling	Children	Child ^a^	21	Likert-type scale (varying)	NR	Patient-specific education handouts with lifestyle recommendations are generated
28. Home Self-Administered Tool for Environmental Assessment of Activity and Diet (HomeSTEAD) [49]	Assess home environment factors related to children’s diet and PA	Children	Parent ^a^	86	5-point Likert scale	Subscale scores	Promotion of healthy feeding practices
29. Lifestyle Behavior Checklist (LBC) [50,51]	Assess parental perceptions and self-efficacy in managing problems related child eating, activity and weight issues	Children with obesity	Parent ^a^	25	Combination of closed and open questions	Subscale scores	NR
30. Pediatric Adapted Liking Survey (PALS) [52]	Assess dietary behaviours linked to caries and obesity risk	Children	Parent ^b^	33	Horizontal visual 5-point Likert scale, (hates it–loves it)	Subscore per domain; ranging from −100 to +100	Tailored motivational diet-related messages for dental caries and obesity prevention
31. Short-Form, Multicomponent Dietary Questionnaire (SF-FFQ4PolishChildren) [53]	Assess dietary and lifestyle behaviours	Children	Child ^a^ or parent ^b^	44	Likert-type scale (varying)	Subscore per domain Cut-offs for low, moderate and high subscores	NR
32. Tool by Hendrie et al. [54]	Assess family activity environment	Children	Parent ^a^	25	5-point Likert scales (strongly disagree–strongly agree)	NR	NR
33. Tool by Huang et al. [55]	Assess correlates of PA and screen time behaviours	Children	Child ^a^ and parent ^a,b^	46	Likert-type scale (varying)	NR	NR
34. Adolescent Lifestyle Profile (ALP) [56,57]	Assess health-promoting behaviours	Adolescents	Child ^a^	42	4-point Likert scale (never-routinely)	Total score; ranging from 42 to 168	NR
35. Childhood Family Mealtime Questionnaire (CFMQ) (reduced) [58]	Assess mealtime environment	Adolescents	Child ^a^	22	5-point Likert scale (never–always)	NR	NR
36. Diet–Lifestyle Index [59]	Assess nutrition and lifestyle quality related to overweight and obesity	Adolescents	Child ^a^	13	Likert-type scale (varying)	Total score; ranging from 11 to 57	NR
37. Shortened Health-Promoting Lifestyle Profile (HPLP) II [60]	Assess health-promoting behaviours	Adolescents	Child ^a^	34	4-point Likert scale (never–routinely)	Subscale scores and total score	NR
38. Tool by Fernald et al. [61]	Assess health behaviour	Adolescents	Child ^a^	16	NR	Total score; ranging from 0 to 3	NR
39. Tool by Hyun et al. [62]	Assess nutrition knowledge	Adolescents	Child ^a^	20	2-point Likert scales (wrong–right)	Total score; ranging from 0 to 20	NR
40. Tool by Hyun et al. [62]	Assess dietary habits	Adolescents	Child ^a^	9	5-point Likert scales (always–never)	Total score; ranging from 0 to 5	NR
41. VISA-TEEN [63]	Assess lifestyle	Adolescents	Child ^a^	11	Combination of closed and open questions	Total score	NR

Note: Tools are sorted by target age. Abbreviations: NR, not reported; y, years. ^a^ Self-reported; ^b^ proxy-reported; ^c^ originally, 18 items were developed, but only 11 were validated.

**Table 2:** Addressed domains and items of lifestyle screening tools for children in the community setting.

Tool Name	Nutrition ^a^	Physical Activity	Sedentary Behaviour/Screen Time	Sleep	Hygiene	Environment	Other
1. NutricheQ [18,19,20]	✓						
2. Toddler Feeding Questionnaire (TFQ) ^b^ [21]	✓						
3. Toddler NutriSTEP ^b^ [22]	✓		Duration of watching TV or using the computer				Growth adequacy, child’s weight status
4. Toddler Dietary Questionnaire (TDQ) [23]	✓						
5. Child Eating Behavior Questionnaire (CEBQ) ^b^ [24]	✓						
6. Nutrition Screening Tool for Every Preschooler (NutriSTEP) [25,26,27]	✓	Frequency of PA	Frequency and duration of watching TV, using computer and playing video games				Parental satisfaction of child’s growth, child’s weight status
7. Preschooler Dietary Questionnaire (PDQ) ^b^ [28]	✓						
8. Preschoolers Diet–Lifestyle Index (PDL-index) [29]	✓	Duration of moderate-to-vigorous PA	Duration of watching TV				
9. Healthy Kids [30]	✓	Preference for playing over watching TV	Duration of watching TV and playing video or computer games	Bedtime			
10. Tool by Das and Ghosh ^b^ [31]	✓						General knowledge on health and lifestyle
11. Start the Conversation 4–12 (STC-4-12) [32]	✓	Frequency and duration of sports, playing outside and being active, barriers and readiness to change regarding PA	Duration of screen time				
12. Healthy Families Survey [33]	✓	Duration of PA, child sees parent being physically active	Duration of watching TV and using other screens, availability of TV in child’s bedroom				
13. Knowledge, Attitudes and Habits (KAH-) questionnaire [34]	✓	Frequency of playing active games, liking exercise, activities after school and during weekends, knowledge and attitudes towards PA	Activities after school and during weekends		Brushing teeth, washing hands, taking bath or shower		Knowledge, attitudes and habits regarding the human body and emotions
14. Parental Self-efficacy Questionnaire [35]	✓	Confidence regarding child being physically active and playing outside	Confidence regarding limiting amount of screen time				
15. Tool by Chacko and Ganesan [36]	✓						
16. Food, Health and Choices questionnaire (FHC-Q) [37]	✓	Frequency of specific activities, medium PA and heavy PA	Frequency and duration of watching TV and playing video games				Self-determination, outcome expectations, self-efficacy, habit strength, goal intention, knowledge and social desirability regarding a healthy lifestyle
17. Healthy Eating and Physical Activity Self-Efficacy Questionnaire for Children (HEPASEQ-C) [38]	✓	Self-efficacy regarding PA					
18. Healthy Eating and Physical Activity Behavior Recall Questionnaire for Children (HEPABRQ-C) [38]	✓	Duration of PA					
19. Eating Behavior Questionnaire for School Children ^b^ [39]	✓				✓ Not further specified	✓ Not further specified	
20. Tool by Drouin and Winickoff [40]	✓				Recent dental care visit		Tobacco smoke exposure
21. Child Nutrition and Physical Activity (CNPA) Screening Tool [41]	✓	Frequency and duration of PA	Duration of media use, availability of media in child’s bedroom				Perception, confidence and importance items on healthy choices
22. Electronic Kids Dietary Index (E-KINDEX) [42]	✓						
23. Family Health Behavior Scale (FHBS) [43]	✓	Duration of being physically active, PA with parents, playing outside, doing sports, preferring indoor activities over outdoor activities, parental PA with child					
24. Family Nutrition and Physical Activity (FNPA) screening tool ^b^ [44,45]	✓	Child’s PA, family PA	Screen time behaviour and monitoring	Sleep duration		Healthy environment	
25. HABITS questionnaire [46]	✓	Frequency of playing outside	Duration of watching TV				
26. Healthy Living for Kids Survey (HLKS) ^b^ [47]	✓	Frequency and duration of ‘hard’, ‘moderate’ and ‘mild’ exercise, frequency of any activity to work up a sweat, self-efficacy for PA	Duration of screen time, number of TV shows/videos watched, self-efficacy for screen time				
27. HeartSmartKids (HSK) ^b^ [48] (HeartSmartKids, LLC, Boulder, US)	✓	Duration of active play or sports	Duration of watching TV and using other screens	✓ Not further specified			Anthropometric measures
28. Home Self-Administered Tool for Environmental Assessment of Activity and Diet (HomeSTEAD) [49]	✓						
29. Lifestyle Behavior Checklist (LBC) [50,51]	✓	Parental problems experiencing and confidence in dealing with child complaining about PA	Parental problem experience and confidence in dealing with child watching too much TV and playing too many computer games				Parental problems experiencing and confidence in dealing with child complaining about problems related to obesity
30. Pediatric Adapted Liking Survey (PALS) [52]	✓				Liking/disliking of brushing teeth, taking a bath, getting dressed		
31. Short-Form, Multicomponent Dietary Questionnaire (SF-FFQ4PolishChildren) ^b^ [53]	✓	Intensity of PA at school and leisure time	Duration of screen time				Family affluence, height, weight
32. Tool by Hendrie et al. [54]		Parental PA involvement, parental opportunity for PA role modelling, parental support of PA	Parental opportunity for screen time role modelling			See domain PA	
33. Tool by Huang et al. [55]		Child’s self-efficacy regarding PA, home PA environment, sports facilities in neighbourhood, family and peer support for PA	Child’s perceived enjoyment of screen-based behaviours with parents, parental role modelling regarding screen time, rules and guidance on screen-based behaviours, availability of electronic screens			Child’s perceived neighbourhood safety, social environment in neighbourhood	
34. Adolescent Lifestyle Profile (ALP) ^b^ [56,57]	✓	At least: frequency and duration of vigorous PA, playing active games with friends					Health responsibility, interpersonal relations, stress management, personal growth
35. Childhood Family Mealtime Questionnaire (CFMQ) (reduced) [58]	✓						
36. Diet–Lifestyle Index [59]	✓	Duration of extracurricular sport activities	Duration of watching TV and playing electronic games				Obesity status of parents
37. Shortened Health-Promoting Lifestyle Profile (HPLP) II ^b^ [60]	✓	✓ Not further specified					Health responsibility, stress management
38. Tool by Fernald et al. ^b^ [61]	✓	At least: frequency and duration of PA	Duration of watching TV				Alcohol use, smoking
39. Tool by Hyun et al. [62]	✓						
40. Tool by Hyun et al. [62]	✓						
41. VISA-TEEN [63]	✓	Duration of moderate and intense PA	Duration of using internet or gaming	Sleep duration	Frequency of brushing teeth and washing hands		Amount of cigarettes smoked, frequency of consuming alcohol and using drugs

Note: Tools are sorted by target age. ^a^ Details on nutrition items are demonstrated in Table 3; ^b^ Specific items of screening tool not fully described.

**Table 3:** Addressed nutrition items of lifestyle screening tools for children in the community setting.

Tool Name	Consumption of Food Groups	Dietary Habits	Psychological Factors Associated with Nutrition	Other
1. NutricheQ [18,19,20]	Vegetables, fruits, milk, dairy products, sweetened beverages, fortified cereals, red meat instead of oily or dark fish, fast food, unhealthy snacks			Age moving to cow’s milk, avoiding foods due to allergy or intolerance
2. Toddler Feeding Questionnaire (TFQ) ^a^ [21]		Parental indulgent and authoritative practices, not further specified		Food environment-related, not further specified
3. Toddler NutriSTEP ^a^ [22]	Vegetables and fruits, flavoured beverages, dairy and substitutes, grains, meat and alternatives, fast food	Eating while watching TV, eating episodes per day, child feeds him- or herself, drinking from bottle with a nipple		Food is expensive, problems with chewing or swallowing when eating, being hungry at mealtimes, child controls amount consumed
4. Toddler Dietary Questionnaire (TDQ) [23]	Vegetables, fruits, dairy, milk beverages, non-milk beverages, grains, white versus non-white bread, meat products, lean red meat, fish, hot potato products, snack products, sweet snacks, spreadable fats, vegemite-type spreads			
5. Child Eating Behavior Questionnaire (CEBQ) ^a^ [24]		Food fussiness, emotional overeating, emotional undereating, satiety responsiveness, slowness in eating, desire to drink, food responsiveness	Enjoyment of food	
6. Nutrition Screening Tool for Every Preschooler (NutriSTEP) [25,26,27]	Vegetables, fruits, dairy, grain products, meat or fish or poultry or alternatives, fast food, supplements	Eating while watching TV, eating episodes per day		Difficulty buying food because of costs, problems with chewing, swallowing, gagging or choking when eating, not hungry because of drinking all day, parental control of amount consumed
7. Preschooler Dietary Questionnaire (PDQ) ^a^ [28]	Vegetables, fruits, dairy, milk beverages, non-milk beverages, grains, white versus non-white bread, meat products, lean red meat, fish, hot potato products, snack products, sweet snacks, spreadable fats, vegemite-type spreads			
8. Preschoolers Diet–Lifestyle Index (PDL-index) [29]	Vegetables, fruits, sweets, dairy products, grains, red meat (products), white meat and legumes, fish and seafood, unsaturated fats			
9. Healthy Kids [30]	Vegetables, fruits, sugar-sweetened beverages, dairy, unhealthy snacks	Parent and child eating together, removing fat from meat		
10. Tool by Das and Ghosh ^a^ [31]				Knowledge on healthy dietary habits, nutrients and child nutrition practice
11. Start the Conversation 4–12 (STC-4-12) [32]	Vegetables and fruits, sugar-sweetened beverages, milk type, unhealthy snacks, fast food		Barriers and readiness to change regarding healthy eating	
12. Healthy Families Survey [33]	Vegetables, fruits, sugar-sweetened beverages, healthy snacks, unhealthy snacks	Eating out, parent and child eating together, picky eating		Parental modelling and parent–child interactions regarding healthy eating, parental food resource management and shopping behaviours
13. Knowledge, Attitudes and Habits (KAH-) questionnaire [34]	Vegetables, fruits, pastries	Consuming breakfast, lunch and dinner, having mid-morning snack, trying new foods	Attitudes towards healthy and unhealthy eating	Knowledge on healthy and unhealthy eating
14. Parental Self-efficacy Questionnaire [35]			Confidence regarding intake of vegetables, fruits, fruit juice, sugary drinks, sweets, dairy, grains, meat and alternatives, sodium, fats and eating out, eating together, child making healthy choices	
15. Tool by Chacko and Ganesan [36]	Vegetables, green leafy vegetables, fruit, cereals, pulses and dahl and non-vegetarian food, milk and coffee and tea and flavoured milk and curd, junk food, food from street shops	Mid-morning and evening snack, meal skipping		
16. Food, Health and Choices questionnaire (FHC-Q) [37]	Vegetables, fruits, sugar-sweetened beverages, processed packaged snacks, fast food		Self-determination, outcome expectations, self-efficacy, habit strength, goal intention, knowledge and social desirability regarding a healthy diet	
17. Healthy Eating and Physical Activity Self-Efficacy Questionnaire for Children (HEPASEQ-C) [38]			Self-efficacy to adhere to recommendations and to choose the healthy option when in temptation	
18. Healthy Eating and Physical Activity Behavior Recall Questionnaire for Children (HEPABRQ-C) [38]	Vegetables, number of colours of vegetables, fruits, soda pop, dairy, healthy snacks	Choosing the healthy option when eating out		
19. Eating Behavior Questionnaire for School Children ^a^ [39]		Food responsiveness, meal timings, eating problems, meal preparation		
20. Tool by Drouin and Winickoff [40]	Sugar-sweetened beverages			
21. Child Nutrition and Physical Activity (CNPA) Screening Tool [41]	Vegetables, fruits, sugar-sweetened beverages, milk, milk type, fast food	Consuming breakfast, dinner eaten with adult	Perception, confidence and importance items on a healthy diet	
22. Electronic Kids Dietary Index (E-KINDEX) [42]	Vegetables, fruits and fruit juices, sweets and junk food, soft drinks, milk, bread, cereals and grain foods, meat, salted and smoked meat food, fish and seafood, legumes, fried food, grilled food	Consuming breakfast, number of main meals and snacks, eating in fast food restaurants or other eating places, eating with family, eating alone, eating of healthy food, eating meals in afternoon school, eating foods because they are advertised, eating whatever food is prepared at home, parental insistence to eat all the food, eating when not hungry	Beliefs and attitudes regarding an (un)healthy diet, weight, dieting	
23. Family Health Behavior Scale (FHBS) [43]		Consuming breakfast, eating three meals a day, eating at table, staying seated at the table, eating at a routine time, asking for unhealthy snacks, eating when bored, emotional eating, eating frequently, sneaking of food	Being influenced to eat or offered unhealthy foods by others	Choices and teaching on healthy foods by parents
24. Family Nutrition and Physical Activity (FNPA) screening tool ^a^ [44,45]	Food choices, beverage choices	Family eating patterns, family eating habits	Restriction/rewarding	
25. HABITS questionnaire [46]	Vegetables, fruits, fruit juice, sugar-sweetened beverages, milk, water, fast food meals, unhealthy snacks	Eating while watching TV, eating three meals a day, eating extra meals or snacks		
26. Healthy Living for Kids Survey (HLKS) ^a^ [47]	Vegetables, fruits, low fat milk, whole wheat bread		Self-efficacy and nutritional intention for healthy eating	
27. HeartSmartKids (HSK) ^a^ [48] (HeartSmartKids, LLC, Boulder, US)	At least: vegetables and fruits, sugar-sweetened beverages (incl. juice), milk, unhealthy snacks	At least: consuming breakfast, eating at restaurants, eating while watching TV		
28. Home Self-Administered Tool for Environmental Assessment of Activity and Diet (HomeSTEAD) [49]		Parent and child eating together at table, eating while TV is on	Parental autonomy support, atmosphere during meals	Parental control and limit setting, eating area decoration
29. Lifestyle Behavior Checklist (LBC) [50,51]			Parental problems experiencing and confidence in dealing with child’s eating habits (e.g., eats too quickly, yells about food, hides food)	
30. Pediatric Adapted Liking Survey (PALS) [52]	^b^ Vegetables, fruits, sugar-sweetened beverages, dairy, meat, fish, beans, peanut butter, unhealthy snacks (sweet, salty and fat)			
31. Short-Form, Multicomponent Dietary Questionnaire (SF-FFQ4PolishChildren) ^a^ [53]	Vegetables, fruits, sugar-sweetened beverages, energy drinks, juices, sweets, dairy, fish, fast food	Breakfast consumption, frequency of having two meals per day		Nutrition knowledge
34. Adolescent Lifestyle Profile (ALP) ^a^ [56,57]	At least: vegetables, fruits, sweets, low fat dairy, chicken or fish instead of beef	At least: consuming breakfast		
35. Childhood Family Mealtime Questionnaire (CFMQ) (reduced) [58]		Mealtime structure, mealtime communication	Family mealtime stress	Appearance weight control
36. Diet–Lifestyle Index [59]	Vegetables, fruits, sweets and added sugars, dairy type, wholegrain, breakfast cereals	Consuming breakfast, eating foods not prepared at home, eating episodes per day, removing visible fat from meat/poultry		
37. Shortened Health-Promoting Lifestyle Profile (HPLP) II ^a^ [60]	NR	NR	NR	NR
38. Tool by Fernald et al. ^a^ [61]	At least: vegetables, fruits			
39. Tool by Hyun et al. [62]				Nutrition knowledge, including general knowledge and knowledge regarding food composition, nutrients and diseases
40. Tool by Hyun et al. [62]	Vegetables, green and orange vegetables, seaweed, fruits, dairy, meat and fish and egg and beans	Consuming breakfast, eating adequate amounts, combining food groups at each meal		
41. VISA-TEEN [63]	Vegetables and fruit, soft drinks, dairy, grains and potatoes, red meats, chicken and fish and eggs, butter and sweets, liquid excluding soft drinks			

Notes: Tools are sorted by target age. We numbered the tools in Table 3 as in Table 1. As tool number 32 and 33 do not describe nutrition items, they have been omitted from Table 3. ^a^ Specific items of screening tool not fully described; ^b^ liking/disliking of food items is used as proxy for intake.

### 3.2. Psychometric Properties

Table 4 demonstrates the validity and reliability outcomes of the included screening tools as illustrated by the different studies. For a total of 39 tools, psychometric properties were evaluated, whereas for two tools [36,61] they were not. The median sample size of the studies showing psychometric properties comprised 277 participants (IQR: 145, 486). Regarding reliability, Cronbach’s α, as a measure of internal consistency, and the intraclass correlation coefficient (ICC), considering test–retest reliability, were assessed for 24 and 11 tools, respectively. Other measures of test–retest reliability, such as Cohen’s kappa (κ, *n* = 4), Pearson’s correlation coefficient (r, *n* = 4) and Spearman’s rho (ρ, *n* = 2), were less evaluated. In general, internal consistency was moderate [64], but due to heterogeneity in the assessed concepts and tool aims, comparison between studies was not appropriate. Test–retest reliability was also highly variable, with eight tools clearly reaching cut-offs for ‘sufficiency’ based on ICC or κ [22,23,25,26,28,31,52,55,63,65]. Regarding validity, features of criterion validity were determined mostly. Criterion validity included sensitivity and specificity (*n* = 6, e.g., to detect nutritional risk or obesity) as well as concurrent validity (*n* = 31, e.g., association of tool score with body mass index (BMI)). Predictive validity was not assessed for any tool. Specifically, the ‘NutricheQ’ was tested for sensitivity, specificity, associations with food group intake and nutrient intake based on a 4-day weighed food diary, and associations with BMI z-scores [18,19,20]. The other screening tools were validated less extensively, usually comprising only one dimension of validity.

**Table 4:** Psychometric properties of lifestyle screening tools for children in the community setting.

Tool Name	Country	Sample Size	Age	Reliability	Criterion Validity
1. NutricheQ	Ireland [18]	*N* = 371	1–3 y	Internal consistency, α = 0.50	Total score was associated with (4-day weighted food diary) intakes of fruits, vegetables, protein, dietary fibre, non-milk sugars, iron, vitamin D, zinc, calcium, riboflavin, niacin, folate, thiamine, phosphorous, potassium, carotene and retinol (r = −0.390–0.119, *p* < 0.05) A score > 4 (AUC = 76%) identified moderate risk with sensitivity = 83% and specificity = 48% A score > 8 (AUC = 85%) identified high risk with sensitivity = 70% and specificity = 80%
	Italy [19]	*N* = 201	1–3 y	Internal consistency, α = 0.83 for Section 1 and α = 0.70 for Section 2 ICC = 0.73 (95% CI [0.40, 0.89], *p* = 0.0002) for Section 1 and ICC = 0.55 (95% CI [0.13, 0.81], *p* = 0.0074) for Section 2	In Section 1, a score ≥ 4 identified toddlers with a poor iron intake (AUC = 0.678, *p* = 0.001) and a score of ≥2 identified toddlers exceeding the En% protein intake (AUC = 0.6024, *p* = 0.009). In Section 2, a score of ≥3 identified toddlers with poor fibre intake (AUC = 0.7028, *p* < 0.0001)
	Lebanon [20]	*N* = 467	1–3 y		Total score was associated with age and BMI (r = 0.11, *p* = 0.021 (for both)), and with fat (ρ = 0.148, *p* = 0.039) and fibre (ρ = −0.137, *p* = 0.031) intake AUC = 0.457 for correctly classifying toddlers into the high risk group based on their BMI z-score
2. Toddler Feeding Questionnaire (TFQ) [21]	United States	*N* = 629	3–5 y	Internal consistency, α = 0.66 for indulgent subscale, α = 0.65 for authoritative subscale, α = 0.48 for environmental subscale	Indulgent subscale scores were correlated with the HEI-2010 (ρ = −0.22, *p* < 0.001), kcal/d (ρ = 0.11, *p* = 0.011), grams of fat/day (ρ = 0.12, *p* = 0.008), servings of vegetables (ρ = −0.11, *p* = 0.01), servings of desserts (ρ = 0.13, *p* = 0.002) and servings of sugary drinks (ρ = 0.23, *p* < 0.001) Authoritative subscale scores were correlated with the HEI-2010 (ρ = 0.15, *p* < 0.001), servings of vegetables (ρ = 0.11, *p* = 0.011), servings of desserts (ρ = −0.15, *p* < 0.001) and servings of sugary drinks (ρ = − 0.09, *p* < 0.039) Environmental subscale scores were correlated with HEI-2010 (ρ = − 0.12, *p* = 0.004), kcals/day (ρ = 0.12, *p* = 0.007), grams of fat/day (ρ = 0.14, *p* = 0.001), servings of desserts (ρ = 0.13, *p* = 0.003) and servings of sugary drinks (ρ = 0.22, *p* < 0.001)
3. Toddler NutriSTEP [22]	Canada	*N* = 200	18–35 m	ICC = 0.951 (*p* < 0.001)	Total score was associated with dietician risk score (ρ = 0.67, *p* < 0.000) A score ≥ 21 identified moderate risk with sensitivity = 86% and specificity = 61% A score ≥ 26 identified high risk with sensitivity = 95% and specificity = 63%
4. Toddler Dietary Questionnaire (TDQ) [23]	Australia	*N* = 111	12–36 m	Total score ICC = 0.90 (*p* < 0.001) All children were classified into the same (*n* = 83, 75%) or adjacent (*n* = 28, 25%) dietary risk category during each administration Test–retest reliability for individual items, κ_w_ = 0.40–0.78	Total score and food frequency questionnaire risk score were associated (r = 0.71, *p* < 0.001) Classification analysis between the TDQ and food frequency questionnaire revealed that all the participants were classified into the same (*n* = 88, 79%) or adjacent (*n* = 23, 21%) dietary risk category
5. Child Eating Behavior Questionnaire (CEBQ) [24]	Sweden	*N* = 1271	3–8 y	Internal consistency: α = 0.73	NR
6. Nutrition Screening Tool for Every Preschooler (NutriSTEP)	Canada [25]	*N* = 269	3–5 y	Total score ICC = 0.89 (95% CI [0.85, 0.92], *p* < 0.001)Test–retest reliability for individual items, κ = 0.39–1.0	Total score was associated with dietician risk rating (r = 0.48, *p* = 0.01) A score > 20 identified moderate risk with sensitivity = 53% and specificity = 79% A score > 25 identified high risk with sensitivity = 84% and specificity = 46%
	Canada [26]	*N* = 63 for internet use *N* = 64 for onscreen use	3–5 y	Internet use total score ICC = 0.91 (95% CI [0.90, 0.96]) Onscreen use total score ICC = 0.91 (95% CI [0.85, 0.95]) Test–retest reliability among risk categories, κ = 0.58 (*p* = 0.000) for internet use and κ = 0.50 (*p* = 0.000) for onscreen use	NR
	Iran [27]	*N* = 192	4–6 y	Test–retest reliability, r = 0.68 (*p* < 0.001)	Total score was associated with nutritionist risk score (r = 0.23, *p* = 0.003) and with healthy eating index (r = −0.16, *p* = 0.03) A score > 27 identified moderate risk with sensitivity = 41.7% and specificity = 85.7% A score > 31 identified high risk with sensitivity = 38.9% and specificity = 84.4%
7. Preschooler Dietary Questionnaire (PDQ) [28]	Australia	*N* = 74	3–5 y	Total score ICC = 0.87 (95% CI [0.07, 2.95], *p* = 0.040)	Total score and food frequency questionnaire risk score were associated (r = 0.85, *p* = 0.009) PDQ scores were associated with the number of people per household (β = −0.32, 95% CI [−6.69, −0.59], *p* = 0.020), but not BMI z-score (β = −0.09, 95% CI [−0.02, −0.04], *p* = 0.512)
8. Preschoolers Diet–Lifestyle Index (PDL-index) [29]	Greece	*N* = 2287	2–5 y	NR	A 1/44 unit score increase was associated with an OR for obesity of 0.95 (95% CI [0.92, 0.98]) and an OR of 0.97 (95% CI [0.95, 0.99]) for overweight/obesity Correct classification rate for obesity = 85%, for overweight/obesity = 67% Sensitivity for obesity = 60%, for overweight/obesity = 55% Specificity for obesity and overweight/obesity = 52%
9. Healthy Kids [30]	United States	*N* = 133	2–5 y	Internal consistency, α = 0.76 Test–retest reliability coefficient = 0.74 (*p* ≤ 0.01)	The Healthy Kids scale score was inversely associated with BMI percentiles (*p* = 0.02)
10. Tool by Das and Ghosh [31]	India	*N* = 134	3–6 y	Internal consistency, α = 0.87 Total score ICC = 0.77 (*p* <0.01)	NR
11. Start the Conversation 4–12 (STC-4-12) [32]	United States	*N* = 115	4–12 y	NR	Three of five queried dietary barriers were found to be significantly associated with at least one healthy eating behaviour Four of five queried barriers to PA were significantly associated with at least one PA-related behaviour
12. Healthy Families Survey [33]	United States	*N* = 1376	6–11 y	Internal consistency for subscales, α = 0.51–0.77	NR
13. Knowledge, Attitudes and Habits (KAH-) questionnaire [34]	Spain	*N* = 295	6–7 y	Internal consistency, α = 0.79	NR
14. Parental Self-efficacy Questionnaire [35]	United States	*N* = 146	6–11 y	Internal consistency, α = 0.94 Test–retest reliability, r = 0.94 (*p* < 0.001)	NR
15. Tool by Chacko and Ganesan [36]	India	NR	6–17 y	NR	NR
16. Food, Health and Choices questionnaire (FHC-Q) [37]	United States	*N* = 221	9–11 y	Internal consistency: α = 0.77–0.92 for behaviour scales and α = 0.44–0.83 for psychosocial scales ICC = 0.59–0.81 for behaviours (*p* < 0.001) and ICC = 0.51–0.68 for continuous psychosocial determinants (*p* <0.05)	Correlation coefficients between the FHC-Q and reference questionnaires were all statistically significant (*p* < 0.01)
17. Healthy Eating and Physical Activity Self-Efficacy Questionnaire for Children (HEPASEQ-C) [38]	United States	*N* = 492	9–13 y	Internal consistency, α = 0.75	HEPASEQ-C was significantly correlated with HEPABRQ-C, r = 0.50 (*p* = 0.000)
18. Healthy Eating and Physical Activity Behavior Recall Questionnaire for Children (HEPABRQ-C) [38]	United States	*N* = 492	9–13 y	NR	HEPABRQ-C was significantly correlated with HEPASEQ-C, r = 0.50 (*p* = 0.000)
19. Eating Behavior Questionnaire for School Children [39]	India	*N* = 462	10–12 y	NR	No correlation between tool subscores and anthropometric measures (exact numerical data NR)
20. Tool by Drouin and Winickoff [40]	United States	*N* = 626	0–18 y	NR	Parents receiving the tool were not more likely to receive counselling or service delivery by clinicians than participants not screened No statistical difference in the proportion of parents reporting having taken steps towards correcting the behaviour in the parents that received the screening after one month follow-up
21. Child Nutrition and Physical Activity (CNPA) Screening Tool [41]	United States	*N* = 2230	2–18 y	Internal consistency: α = ‘low’, exact value NR	Both generated readiness to change and perception subscores were associated with weight status categories (*p* < 0.001)
22. Electronic Kids Dietary Index (E-KINDEX) [42]	Greece	*N* = 622	9–13 y	Internal consistency: α = 0.60	Each 1 SD (i.e., 7.81 points) score increase was associated with a 2.31 ± 0.23 kg/m^2^ decrease in BMI (*p* < 0.001), a 2.23 ± 0.35 decrease in calculated % body fat (*p* < 0.001) and a 2.16 ± 0.61 cm decrease in waist circumference (*p* < 0.001) Correct classification rate for excess body fat was 84% (95% CI [0.74, 0.94]) Sensitivity for overweight/obesity versus normal weight = 74%, for obesity versus normal weight/overweight = 61% Specificity for overweight/obesity versus normal weight = 46%, for obesity versus normal weight/overweight = 79%
23. Family Health Behavior Scale (FHBS) [43]	United States	*N* = 233	5–12 y	Internal consistency: α = 0.86 Test–retest reliability coefficient = 0.85	FHBS was inversely associated with zBMI (r = −0.28, *p*< 0.01) Every unit increase was associated with an OR of 0.96 (95% CI [0.95, 0.99] for overweight/obesity (*p* < 0.01) Correct classification rate for weight classification = 63%
24. Family Nutrition and Physical Activity (FNPA) screening tool	United States [44]	*N* = 349	1st and 10th grade	NR	At both ages, the FNPA score was not significantly correlated with BMI% Only in first graders, scores in the lowest tertile were associated with higher odds for overweight/obesity compared to the highest tertile (OR = 2.49, 95% CI [1.17, 5.31])
	United States [45]	*N* = 19	2–5 y	NR	NR
25. HABITS questionnaire [46]	United States	*N* = 35	7–16 y	Internal consistency, α = 0.61 for dietary subscale and α = 0.59 for PA/sedentary behaviour subscale Test–retest reliability, κ = 0.27–0.78 for individual items of dietary subscale and κ = 0.29–0.48 for PA/sedentary behaviour subscale As a whole, the dietary subscale and PA/sedentary behaviour subscales had test–retest reliabilities of r = 0.94 and r = 0.87, respectively	NR
26. Healthy Living for Kids Survey (HLKS) [47]	United States	*N* = 88	9–12 y	Internal consistency for subscales, α = 0.63–0.80 Test–retest reliability for subscales: r = 0.37–0.78	NR
27. HeartSmartKids (HSK) [48] (HeartSmartKids, LLC, Boulder, US)	United States	*N* = 103	9–14 y	Test–retest reliability, ρ = 0.38–0.78	Each item of the HSK was significantly correlated with the HABITS, ρ = 0.21–0.65 (*p* <0.05)
28. Home Self-Administered Tool for Environmental Assessment of Activity and Diet (HomeSTEAD) [49]	United States	*N* = 129	3–12 y	Internal consistency for subscales, α = 0.62–0.93 Subscale ICC = 0.57–0.89	No statistically significant correlation between factor composite scores and child BMI z-scores
29. Lifestyle Behavior Checklist (LBC)	Australia [50]	*N* = 182	4–11 y	Internal consistency, α = 0.97 for Problem scale and α = 0.92 for Confidence scale Test–retest reliability, ρ = 0.87 (*p* < 0.001) for Problem scale and ρ = 0.66 (*p* < 0.001) for Confidence scale	Correct classification rate for obesity was 91%
	The Netherlands [51]	*N* = 273	3–13 y	Internal consistency, α = 0.92 for Problem scale and α = 0.98 for Confidence scale Test–retest reliability, ρ = 0.74 (*p* < 0.001) for Problem scale and ρ = 0.70 (*p* < 0.001) for Confidence scale	Parents with healthy weight children scored lower on the Problem scale, F = 16.94 (*p* < 0.001), compared to those with overweight children The Problem scale was associated with nurturance (ρ = −0.23, *p* < 0.01), restrictiveness (ρ = 0.14, *p* < 0.05), psychological control (ρ = 0.19, *p* < 0.01) and BMI of child (ρ = 0.21, *p* < 0.01), mother (ρ = 0.23, *p* < 0.01) and father (ρ = 0.14, *p* < 0.05) The Confidence scale was associated with nurturance (ρ = 0.14, *p* < 0.05) and psychological control (ρ = −0.22, *p* < 0.01)
30. Pediatric Adapted Liking Survey (PALS) [52]	United States	*N* = 144	5–17 y	Internal consistency for subscales, α = 0.40–0.72 ICC for individual items = 0.79–0.91	In girls, higher BMI was associated with greater preference for fat/sweet/salty foods (β = 0.32, 95% CI [0.14, 1.15], *p* < 0.05)
31. Short-Form, Multicomponent Dietary Questionnaire (SF-FFQ4PolishChildren) [53]	Poland	*N* = 437 children *N* = 630 adolescents	6–10 y 11–15 y	Test–retest reliability for consumption of food items and meals, κ = 0.46–0.81 in children, κ = 0.30–0.54 in adolescent’s test–retest, and κ = 0.27–0.56 in adolescent’s test and parent’s retest Across study groups, test–retest reliability, κ = 0.31–0.72 for active/sedentary lifestyle items, κ = 0.55–0.93 for components of the Family Affluence Scale, κ = 0.64–0.67 for BMI categories, κ = 0.36 for the nutrition knowledge of adolescents and κ = 0.62 for the nutrition knowledge of children’s parents	NR
32. Tool by Hendrie et al. [54]	Australia	*N* = 106	5–11 y	Internal consistency, α = 0.83	The family activity environment was associated with children’s fruit and vegetable intake assessed with a 24-h recall (r = 0.34, *p* < 0.01), PA assessed by the Children’s Leisure Activity Study Survey (r = 0.27, *p* < 0.01) and screen time (r = −0.24, *p* < 0.05) assessed by a survey
33. Tool by Huang et al. [55]	China	*N* = 303	9–14 y	Internal consistency for identified factors, α = 0.50–0.86 Identified factor ICC = 0.82–0.89	Self-efficacy (r = 0.25, *p* < 0.05), home physical activity environment (r = 0.14, *p* < 0.05) and peer support (r = 0.25, *p* < 0.05) were associated with child-reported moderate-to-vigorous PA Family support for PA was associated with screen time (r = −0.22, *p* < 0.05)
34. Adolescent Lifestyle Profile (ALP)	United States [56]	*N* = 207	10–15 y	Internal consistency: α = 0.91	ALP correlated with hope (r = 0.60, *p* = 0.001), self-efficacy (r = 0.47, *p* = 0.001) and self-esteem (r = 0.35, *p* = 0.001) scores
	Portugal [57]	*N* = 236	12–18 y	Internal consistency: α = 0.87	NR
35. Childhood Family Mealtime Questionnaire (CFMQ) (reduced) [58]	United States	*N* = 280	11–15 y	Internal consistency for identified factors, α = 0.76–0.82	Childhood mealtime communication was associated with physically active days (β = 0.20, 95% CI [0.07, 0.32], *p* < 0.01), fruits and vegetable intake (β = 0.29, 95% CI [0.15, 0.45], *p* < 0.001) and added sugar intake (β = 0.23, 95% CI [0.09, 0.37], *p* < 0.001) Childhood mealtime stress was associated with fruits and vegetable intake (β = 0.26, 95% CI [0.08, 0.45], *p* < 0.01) and added sugar intake (β = 0.38, 95% CI [0.21, 0.57], *p* < 0.001)
36. Diet–Lifestyle Index [59]	Greece	*N* = 2008	12–17 y	NR	The Diet–Lifestyle Index was inversely associated with BMI in boys (ρ = −0.169, *p* < 0.001) and girls (ρ = −0.143, *p* < 0.001) An 11/57 unit score increase was associated with an OR of 0.93 (95% CI [0.90, 0.96]) for overweight/obesity (*p* < 0.001) Correct classification rate for BMI category = 83% Sensitivity for overweight/obesity = 66%, specificity = 50%
37. Shortened Health-Promoting Lifestyle Profile (HPLP) II [60]	Iran	*N* = 495	14–18 y	Internal consistency, α = 0.86	Total HPLP-II was associated with quality of life (r = 0.24, *p*< 0.001), self-efficacy (r = 0.48, *p* < 0.001) and demographic variables (data NR)
38. Tool by Fernald et al. [61]	United States	*N* = 227	Average 15 y	NR	NR
39. Tool by Hyun et al. [62]	Korea and China	*N* = 406	15–18 y	NR	Nutrition knowledge was associated with body shape satisfaction in Korean boys (r = 0.208, *p* < 0.01), not in Chinese boys
40. Tool by Hyun et al. [62]	Korea and China	*N* = 406	15–18 y	NR	Healthy dietary habits were associated with body shape satisfaction in Chinese boys (r = 0.210, *p* < 0.01), not in Korean boys
41. VISA-TEEN [63]	Spain	*N* = 419	13–19 y	Internal consistency, α = 0.66 Total score ICC = 0.86 (95% CI [0.82, 0.89])	Total VISA-TEEN score was associated with KIDSCREEN-10 (r = 0.21, *p* < 0.001) and self-rated health (*p* < 0.001)

Note: Tools are sorted by target age. Abbreviations: y, years; ICC, intraclass correlation coefficient; NR, not reported; HEI-2010, Healthy Eating Index 2010.

### 3.3. Implementation

A total of 35 tools calculated a subscore and/or total score. Six tools defined score cut-offs for the identification of risk [18,19,20,22,23,25,26,27,28,53]. Eighteen tools provided some form of a prospect of action following the answers given. Two of these tools [32,40] based their prospects of action on highlighted topics, whereas the other sixteen based prospects of action on tool scores. None of the tools for adolescents provided a prospect of action. The prospects of action could be intended for the health care professional, child or parent. It included counselling, education, a combination of these two, initiating the conversation about a healthy lifestyle or referring to a specialist for further examination, and/or treatment. Articles on the ‘NutriSTEP’, ‘Start the Conversation 4–12′, ‘tool by Drouin and Winickoff’, ‘HeartSmartKids’ (HeartSmartKids, LLC, Boulder, US) and ‘Pediatric Adapted Liking Survey’ described that their prospects of action are tailored to the answers given, but details on them were lacking [25,26,27,32,40,48,52]. The ‘NutricheQ’ was advised to be administered during regular growth check-ups [18,19,20]. Other tools did not describe recommendations for administering occasion or frequency. Despite being developed for out-of-hospital use, the intended target location of administering the tools was merely suggested. When administration methods were reported, it involved paper (*n* = 15) or online (*n* = 10) formats. The ‘NutriSTEP’ paper version was expanded by an internet and onscreen version in response to the interest of health care professionals [26] and the ‘Food, Health and Choices questionnaire’ used an audience response system to decrease administer burden [37]. Others did not describe their motivation for the choice of administration methods.

## 4. Discussion

The 41 lifestyle screening tools for children included in this review varied widely in their design, but items on nutrition, PA and sedentary behaviour/screen time were commonly addressed. Nutrition items predominantly covered the intake of specific food groups, dietary habits and psychological factors, such as (parental) beliefs and attitudes towards a healthy lifestyle. For most tools, one or more aspects of reliability and/or validity had been studied with varying results. Nearly half of the screening tools offered prospects of action, but none described the exact follow-up actions based on tool outcomes. Moreover, other features of implementation were sparse. 

Most tools evaluated lifestyle determinants related to overweight and obesity. Considering overweight, domains related to energy balance, i.e., nutrition, PA and sedentary behaviour, were frequently evaluated. Compared to PA and sedentary behaviour/screen time, which mainly concerned frequency and duration, there was more variety in nutrition items, which reflects the versatility of this topic. The tools not only addressed the intake of foods directly related to energy intake, such as sugar-sweetened beverages and unhealthy snacks/fast food but also foods and dietary habits that might be more indirectly associated with weight status, such as fruits and vegetables, having breakfast and eating together at the table [66,67,68]. The concept of a balanced diet, characterised by adequate amounts and proportions of nutrients required for good health, is broader than energy balance alone. The ‘NutricheQ’ aimed to evaluate the risk of dietary imbalances in toddlers, with a particular focus on iron and vitamin D [18,19,20]. Next to iron and vitamin D, the total score of the ‘NutricheQ’ was associated with the intake of fruits, vegetables, protein, dietary fibre, non-milk sugars and other specific micronutrients [18], and its 18-item version score was also associated with BMI z-scores [20], indicating extensive dietary exploration. It could be proposed that screening tools addressing both dietary and energy balance may be most effective in screening for the risk of overall health problems, including overweight. This could for instance be conducted through the assessment of children’s adherence to age-specific recommendations for commonly consumed food groups. 

While there is emerging evidence on the importance of sleep on weight status and overall health [69,70], only four tools covered sleep. This finding accords with the results of Byrne et al., who conducted a systematic review on brief tools measuring obesity-related behaviours for children under five years of age [17]. Only two out of their twelve appraised tools covered sleep, indicating paucity [17]. Regarding the specific items on sleep, sleep duration was the most common in our results. A systematic review on sleep and childhood obesity supports the relevance of sleep duration on weight status but stated that associations with other dimensions, such as sleep quality and bedtime, need to be studied further [69]. The previous findings that shorter sleep duration in children is associated with unhealthy dietary habits and lower PA suggest a pathway from sleep deficiency to obesity and indicate that certain lifestyle behaviours might cluster in individuals [71,72]. 

The ten screening tools specifically developed for toddlers and preschoolers covered fewer domains than the tools for the other age groups; yet, all comprised nutrition. The early years of life form a critical window of opportunity for growth and development, in which proper nutrition is fundamental [1]. However, other lifestyle factors, such as PA, sedentary behaviour and sleep, have also been shown to affect health in toddlers and preschoolers [5,6,7]. An explanation for the lack of these domains in tools for toddlers and preschoolers might be that guidelines on these topics for this age group are not universally available. Howbeit, none of the reviewed articles clearly justified their choice of the exact items included. Depending on the aim of the lifestyle screening tool, it could be useful to base tool domains on clustering lifestyle behaviours in the target population to provide integrated follow-up advice. In addition, it might be valuable to study accurate indicators of an unhealthy lifestyle in advance. Furthermore, the accuracy of the questions should be optimized to obtain the desired information (e.g., the exact question to evaluate general vegetable intake). 

In addition to lifestyle behaviours and habits, the included screening tools evaluated psychological factors related to lifestyle. Psychological factors, such as parental attitudes towards healthy eating and self-efficacy to adhere to recommendations, are important [73]. On the one hand, these perceptions can imply certain behaviours. On the other, they can map motivation and perceived barriers for behaviour change. As children’s lifestyle behaviour is highly reliant on parental support behaviours [74], it is helpful to evaluate parental perceptions regarding lifestyle. When health care professionals gain an insight into parental indicators of behaviour change, they obtain cues for motivational interviewing to help parents and children shifting towards a healthier lifestyle. 

Although 39 out of 41 screening tools had undergone some form of psychometric testing, the results were inconclusive and hardly comparable due to high heterogeneity in tool aim and study design. However, a number of tools, such as the ‘NutricheQ’, ‘NutriSTEP’ and Lifestyle Behavior Checklist [18,19,20,25,26,27,50,51], have been researched more thoroughly than others and may therefore have a more solid foundation for use in practice. Becker et al. [14] concluded in their review that no nutrition screening tool for children in the community setting provided enough evidence for moderate to high validity and reliability [14]. As the reliability and validity influence the effectiveness of screening tools, assessing these psychometric properties is crucial. Nevertheless, the interpretation of group-level validity and reliability for individual counselling should be performed with prudence [75]. Proper psychometric assessment should also take into account differences in socioeconomic status and language and fill the current gap in testing predictive validity. The lack of a gold standard for screening children’s lifestyle impairs the validity testing of new lifestyle screening tools. Nonetheless, studying the association of validated dietary assessment methods and activity trackers with items of lifestyle screening tools could assess criterion validity. In addition, longitudinal studies addressing a common outcome of an unhealthy lifestyle, such as overweight, and applying identical intervention strategies could study the effectiveness of a new tool over another one or over a health care professional’s clinical view. 

Eighteen tools provided recommendations for actions to be taken based on the answers given. Overall, these recommendations for both children and parents were as general as ‘receiving tips’ or health care professionals ‘offering counselling’ or ‘referring to a specialist’, and are therefore open to interpretation. Neither of the tools that identified cut-offs for particular risk classifications defined clear follow-up actions according to the classification. This is in contrast with established nutrition screening tools for hospitalised children, which offer specific action points per identified risk group [76,77,78,79]. Defining risk score cut-offs corresponding with unambiguous follow-up steps, such as ‘no action required’, ‘discuss lifestyle with parents and repeat screening in X weeks’ and ‘initiate further examination by a specialist’, might strengthen the effectiveness of lifestyle screening tools. Considering the various domains of lifestyle, integrating subscores and cut-offs for different domains could pinpoint the areas that need attention and guide health care professionals to address these specifically. 

With this review, we have created a hitherto lacking overview of the literature. Searching for screening tools encompassing lifestyle in the broadest sense of the term made our search strategy comprehensive and enabled the inclusion of tools that evaluate a broad variety of lifestyle determinants. Our additional focus explicitly on nutrition highlighted the importance of this topic within children’s lifestyle. 

Not preselecting specific lifestyle factors (except nutrition) in our search strategy could also be considered a limitation, as we may have missed articles on screening tools that only denote specific determinants (e.g., PA and screen time), without framing them in the context of lifestyle in general. Moreover, we might have missed certain screening tools due to publication bias. Another important concern was the definition of screening tools, which we predefined in our protocol as tools that assign a certain value to behaviour and/or characteristics and/or offer prospects of action to an individual. The ascertainment of screening tools was performed in duplicate and independently, but the lack of a universal definition may have hampered the robustness of our methods. As this review was conducted to provide an overview of all recent literature on lifestyle screening tools for children in the community setting, regardless of methodological quality and tool outcome, we did not include a quality or risk of bias assessment. However, we expect that the limitations of this review have not altered the main conclusions and that we gained clear insights into existing lifestyle screening tools for children. 

Ideally, a balance exists between the set of items retrieving as much information as possible and convenience by the person completing the tool. Considering the association between questionnaire length and response burden [80], future studies should target the optimal number of items relative to the aim of the screening tool. Moreover, addressing aspects of implementation of a screening tool might contribute to fulfilling the potential of its usage. For example, studies that explore the most effective administration method (e.g., paper format, online or mobile application), setting (e.g., at home or at a clinic) and target group of health care professionals handling the results of the screening tool could detect vital features in making the screening tool advantageous. Finally, it is crucial to validate current and new lifestyle screening tools to identify children at risk as early as possible.

## 5. Conclusions

This systematic review shows that a fair variety exists in lifestyle screening tools for children in the community setting. The majority addressed dietary and/or lifestyle behaviours and habits related to overweight and obesity. Domains that were mostly covered included nutrition, PA and sedentary behaviour/screen time. Tool validation was, however, limited, and the availability of unambiguous prospects of actions following tool outcomes was lacking. Considering the importance of a healthy lifestyle during childhood, there is a need for an easy-to-administer lifestyle screening tool for children with distinct follow-up actions in order to improve a child’s lifestyle at an early age.

## Data Availability

Not applicable.

## Supplementary Materials

The following supporting information can be downloaded at: https://www.mdpi.com/article/10.3390/nu14142899/s1, Supplementary file S1: Search strategy.
