# Supplementary material for: Lifestyle Screening Tools for Children in the Community Setting: A Systematic Review
Source: Nutrients. 2022 Jul 14;14(14):2899. doi: 10.3390/nu14142899 (PMC9325265; doi:10.3390/nu14142899)
Supplement: Supplementary file 1 [file nutrients-14-02899-s001.zip › nutrients-1802755-supplementary.pdf]

Supplementary file S1: Search strategy

All searches were performed on July 27, 2020.

Searches lifestyle tools

PubMed:

Search	Search terms	Number of hits
#1	“child”[mh] OR “infant”[mh] OR “adolescent”[mh] OR “pediatrics”[mh] OR child*[ti] OR infant*[ti] OR adolescent*[ti] OR pediatric*[ti] OR paediatric*[ti] OR toddler*[ti] OR preschool*[ti] OR youth*[ti]	3,777,091
#2	“Surveys and Questionnaires”[mj] OR “Mass Screening”[mj] OR screening[ti]	330,386
#3	“life style”[mj] OR lifestyle*[ti] OR life-style*[ti]	45,048
#4	#1 AND #2 AND #3	552
#5	#1 AND #2 AND #3 Filters: Humans, Dutch, English, from 2004 - 2020	404

Cinahl:

Search	Search terms	Number of hits
#1	MH “Child+” OR MH “Infant+” OR MH “Adolescence+” OR MH “Pediatrics+” OR TI(child* OR infant* OR	1,090,303

	adolescent* OR pediatric* OR paediatric* OR toddler* OR preschool* OR youth*)	
#2	MM “Questionnaires+” OR MM “Surveys+” OR MM “Health Screening+” OR TI(screening)	97,764
#3	MM “Life Style+” OR TI(Lifestyle* OR Life-style*)	122,915
#4	#1 AND #2 AND #3	540
#5	#1 AND #2 AND #3 Filters: Humans, from 2004-2020	372

Embase:

Search	Search terms	Number of hits
#1	‘child’/exp OR ‘infant’/exp OR ‘adolescent’/exp OR ‘pediatrics’/exp OR child*:ti OR infant*:ti OR adolescent*:ti OR pediatric*:ti OR paediatric*:ti OR toddler*:ti OR preschool*:ti OR youth*:ti	4,022,363
#2	‘questionnaire’/exp/mj OR ‘health survey’/exp/mj OR ‘survey’/exp/mj OR ‘screening’/exp/mj OR screening:ti	377,246
#3	‘lifestyle’/exp/mj OR lifestyle*:ti OR life-style*:ti	33,889
#4	#1 AND #2 AND #3	176
#5	#1 AND #2 AND #3 Filters: Humans, Dutch, English, from 2004 - 2020	123

Updates Becker et al. [14]

PubMed

Search	Search terms	Number of hits
#1	"nutritional risk"[Title/Abstract] OR "malnutrition risk"[Title/Abstract] OR "nutrient poor"[Title/Abstract] OR "dietary risk"[Title/Abstract] OR "child nutrition disorders/etiology"[MeSH Terms] OR "feeding and eating disorders of childhood/diagnosis"[MeSH Terms] OR "feeding and eating disorders of childhood/etiology"[MeSH Terms] OR "malnutrition/diagnosis"[MeSH Terms] OR "Nutrition Assessment"[MeSH Terms] OR "child nutrition disorders/diagnosis"[MeSH Terms] OR "Nutrition Assessment"[Title/Abstract] OR "nutrition diagnostic"[Title/Abstract] OR "nutrition diagnosis"[Title/Abstract] OR "nutrition screening"[Title/Abstract] OR "malnutrition assessment"[Title/Abstract] OR "malnutrition-inflammation score"[Title/Abstract] OR "Malnutrition/diagnosis"[Title/Abstract] OR "malnutrition diagnostic"[Title/Abstract] OR "malnutrition screening"[Title/Abstract] OR "nutritional assessment"[Title/Abstract] OR "nutritional diagnosis"[Title/Abstract] OR "nutritional screening"[Title/Abstract]	36,983
#2	"Child"[MeSH Terms] OR "Adolescent"[MeSH Terms] OR "Pediatrics"[MeSH Terms] OR "Child"[Title/Abstract] OR	3,737,556

	"children"[Title/Abstract] OR "Adolescent"[Title/Abstract] OR "adolescents"[Title/Abstract] OR "pediatric"[Title/Abstract] OR "paediatric"[Title/Abstract] OR "paediatrics"[Title/Abstract] OR "Pediatrics"[Title/Abstract] OR "infant"[Title/Abstract] OR "infants"[Title/Abstract]	
#3	"reliability"[Title/Abstract] OR "sensitive"[Title/Abstract] OR "sensitivity"[Title/Abstract] OR "specific"[Title/Abstract] OR "specificity"[Title/Abstract] OR "validated"[Title/Abstract] OR "validation"[Title/Abstract] OR "validity"[Title/Abstract] OR "Sensitivity and Specificity"[MeSH Terms] OR "Reproducibility of Results"[MeSH Terms] OR "Reference Values"[MeSH Terms] OR "overall agreement"[Title/Abstract] OR "detected"[Title/Abstract] OR "correlated"[Title/Abstract]	6,062,749
#4	#1 AND #2 AND #3 NOT (animals[mh] NOT humans[mh]) AND English[la] AND 2017/01/01:2020/07/27[pdat]	558

Cinahl

**Search**	**Search terms**	**Number of hits**
#1	MH "Child Nutrition Disorders+" OR MH "Infant Nutrition Disorders/DI/ET" OR MH "Feeding and Eating Disorders of Childhood/DI/ET" OR MH "Malnutrition/DI/ET" OR MH "Nutritional Assessment"	18,495
#2	TI ((nutrition* or malnutrition or dietary) n1 (risk* or diagnos* or screen* or assessment*))	3,252

#3	TI (Nutrient* n1 poor)	28
#4	TI “malnutrition-inflammation score”	23
#5	AB ((nutrition* or malnutrition or dietary) n1 (risk* or diagnos* or screen* or assessment*))	7,003
#6	AB (Nutrient* n1 poor)	278
#7	AB “malnutrition-inflammation score”	75
#8	MH "Child+" OR MH "Infant+"	670,219
#9	TI (Child* OR adolescen* OR pediatric* OR paediatric* OR infant*)	467,626
#10	AB (Child* OR adolescen* OR pediatric* OR paediatric* OR infant*)	509,052
#11	MH "Reference Values" OR MH "Reproducibility of Results" OR MH "External Validity" OR MH "Reliability+" OR MH "Reliability and Validity+"	308,452
#12	TI (reliability OR sensitive OR sensitivity OR specific OR specificity OR validated OR validation OR validity)	99,472
#13	AB (reliability OR sensitive OR sensitivity OR specific OR specificity OR validated OR validation OR validity)	546,007
#14	#1 OR #2 OR #3 OR #4 OR #5 OR #6 OR #7	23,730
#15	#8 OR #9 OR #10	953,415
#16	#11 OR #12 OR #13	772,646

#17	#14 AND #15 AND #16 Filter: published between 2017-2020	381

Embase

Search	Search terms	Number of hits
#1	'child'/exp OR 'adolescent'/exp OR 'infant'/exp	3,724,335
#2	child*:ti,ab OR adolescen*:ti,ab OR pediatric*:ti,ab OR paediatric*:ti,ab OR infant*:ti,ab	2,568,360
#3	‘sensitivity and specificity’/exp OR ‘reproducibility’/exp OR ‘reference value’/exp OR ‘validity’/exp OR ‘reliability’/exp	876,173
#4	reliability:ti,ab OR sensitive:ti,ab OR sensitivity:ti,ab OR specific:ti,ab OR specificity:ti,ab OR validated:ti,ab OR validation:ti,ab OR validity:ti,ab	5,340,083
#5	((nutrition* OR malnutrition OR dietary) NEAR/1 (risk* OR diagnos* OR screen* OR assessment*)):ti,ab	18,600
#6	(nutrient* NEAR/1 poor):ti,ab	1,769
#7	'malnutrition-inflammation score':ti,ab	325
#8	'nutritional assessment'/exp	30,493
#9	'nutritional status'/exp	67,730
#10	#1 OR #2	4,411,762
#11	#3 OR #4	5,691,695

#12	#5 OR #6 OR #7 OR #8 OR #9	98,617
#13	#10 AND #11 AND #12	5,197
#14	#13 AND [English]/lim AND [2017-2020]/py	1,425

Update exploratory systematic search

PubMed

Search	Search terms	Number of hits
#1	"infant"[mh] OR "child"[mh] OR "adolescent"[mh] OR infant[tiab] OR infants[tiab] OR infanthood[tiab] OR preschool[tiab] OR preschooler[tiab] OR toddler[tiab] OR toddlers[tiab] OR toddlerhood[tiab] OR child[tiab] OR children[tiab] OR childhood[tiab] OR adolescent[tiab] OR adolescents[tiab] OR adolescent[tiab] OR adolescents[tiab] OR adolescence[tiab] OR teen[tiab] OR teens[tiab] OR teenager[tiab] OR teenagers[tiab] OR youth[tiab] OR youths[tiab]	4,003,592
#2	"malnutrition"[mh:noexp] OR "overnutrition"[mh:noexp] OR malnutrition[tiab] OR malnourished[tiab] OR malnourishment[tiab] OR malnutrition risk[tiab] OR undernutrition[tiab] OR overnutrition[tiab] OR dietary imbalance[tiab] OR dietary imbalances[tiab] OR nutrition imbalance[tiab] OR nutritional imbalance[tiab] OR nutrition imbalances[tiab] OR nutritional imbalances[tiab] OR nutrition risk[tiab] OR nutritional risk[tiab]	58,551

#3	“mass screening”[mh:noexp] OR “nutrition assessment”[mh] OR screening tool[tiab] OR screening tools[tiab] OR screening instrument[tiab] OR screening instruments[tiab] OR nutrition questionnaire[tiab] OR nutritional questionnaire[tiab] OR nutrition screening[tiab] OR nutritional screening[tiab] OR nutrition risk screening[tiab] OR nutritional risk screening[tiab] OR malnutrition risk screening [tiab] OR nutrition assessment[tiab] OR nutritional assessment[tiab] OR dietary assessment[tiab]	145,375
#4	#1 AND #2 AND #3 AND 2019/01/01:2020/07/27[pdat] Filters: Dutch, English, Humans	80
